# Dental Laboratory Production of Prosthetic Restorations in a Population in Sofia, Bulgaria: A Descriptive Study 

**DOI:** 10.1155/2010/286192

**Published:** 2010-12-21

**Authors:** Nikola D. Damyanov, Dick J. Witter, Anneloes E. Gerritsen, Nico H. J. Creugers

**Affiliations:** ^1^Department of Prosthetic Dental Medicine, Faculty of Dental Medicine, Medical University-Sofia, 1, Georgi Sofiiski Boulevard, 1431 Sofia, Bulgaria; ^2^Department of Oral Function and Prosthetic Dentistry, College of Dental Science, Radboud University Nijmegen Medical Centre, P.O. Box 9101, 6500HB Nijmegen, The Netherlands

## Abstract

*Objective*. To describe prosthodontic production related to mutilated dentitions in Sofia, Bulgaria. *Methods*. Prosthodontic production from 5 dental laboratories was recorded during a 14-day period. Production was related to dentitions as noted from casts. Dentitions were classified as edentulous, interrupted/reduced, slightly interrupted, shortened, and complete. The representativeness of the laboratory sample was verified trough comparison with a Sofia population sample using proportions of crowned or replaced teeth per dental region. *Results*. The total production consisted of 243 crowns, 16 post and cores, 82 fixed dental prostheses, and 41 removable dentures. Proportions of crowned teeth were significantly different between the samples; proportions of replaced teeth were not. Of the 58 incomplete dentitions analyzed, 19 were restored to the level of completeness, 15 resulted in slightly interrupted, and 24 in shortened dentitions. *Conclusions*. Predominantly fixed restorations were provided to restore mutilated dentitions to a functional level and not necessarily to complete dentitions.

## 1. Introduction

The demand for restorative treatment is generally triggered by various oral conditions that affect masticatory performance, appearance, and psychological comfort [1]. When tooth replacement is indicated, clinicians must decide which of the available prosthetic restorations will meet patients' demands at best. These restorations are fixed and removable dental prostheses retained and supported by either natural teeth or dental implants. As dental implants are not affordable for a vast majority of patients, the choice is often limited to conventional tooth supported prostheses. In general, fixed dental prostheses are preferred as they offer better function and acceptance [2]. Nevertheless, when several teeth are missing and financial means are limited, removable partial dentures might be indicated. Thus, the decision-making process is usually based on numerous clinical, subjective, and economic considerations related to prevailing health care systems [3, 4]. 

In Bulgaria, the health care system suffers from considerable financial inadequacy with a total expenditure on health care of 4.3% of the gross domestic product (*€*132 per capita per year including dentistry) [5, 6]. Financial limitations in the health care system, together with other factors, are expected to have a negative impact on the oral health of the population. The scarce available data on the oral health of the Bulgarian population indicated high prevalence of missing permanent teeth ranging from 1.3 (20–24 years age group), through 5.3 (35–44 years age group), to 13 (55–64 years age group) [7]. Since the prevalence of missing teeth is substantial and (oral) health budget is restricted, it is crucial that viable and appropriate management strategies, such as the shortened dental arch concept, are utilized [8]. 

Being a minimal intervention approach, the shortened dental arch concept advocates for a “wait and see” period of monitoring function and stability of the dentition instead of immediate replacement of absent molars [9]. Replacement of absent molars with the sole purpose to restore dental arch morphology irrespective of the degree of functional impairment may be considered overtreatment. 

Data on the prevalence of missing teeth, that indicate the need for prosthodontic services, are generally available. The same is not true for data on the provision of prosthetic restorations. The latter can be used to appraise the effective demand for prosthodontic care and to determine utilizable treatment modalities. As part of a larger comprehensive epidemiological study on oral function in reduced dentitions and the feasibility of the shortened dental arch concept within the existing health care system in Bulgaria, the purpose of the present study was to explore prosthodontic production as delivered by dental laboratories.

## 2. Material and Methods

### 2.1. Laboratory Sample

Five commercial dental laboratories in the city of Sofia participated in the study. One of the laboratories was considered small (2 technicians) and four were of average size (4 to 10 technicians). Output from these laboratories was considered representative for Sofia because their clientele (10 to 40 dental practitioners per laboratory) practiced widely in the city. The chief dental technician of each laboratory was asked to record structured information regarding the status of teeth from gypsum casts and all restorations delivered during a two-week period. Additionally, age and gender of patients as reported by the dentists in charge and cost of each restoration, produced were recorded. The teeth of the gypsum casts were described as absent, present (including existing fixed replacements), crown preparation/abutment, or as tooth root. After accomplishment of the restoration the presence or absence of occlusal contact for each tooth was recorded, as well. For relating prosthetic restorations with dental arch and dentition conditions, only sets of complete upper and lower casts were considered. Dental arches (representing either mandible or maxilla) were classified as edentulous, interrupted (2 groups: interrupted/reduced and slightly interrupted), shortened, or complete based on number and type of teeth (Table 1). Dentitions (representing mandible plus maxilla) were classified as edentulous (2 groups: one jaw edentulous and both jaws edentulous), interrupted (2 groups: interrupted/reduced and slightly interrupted), shortened, or complete based on number and type of teeth, and occluding regions (Table 1). Complete sets of casts were assigned according to this classification before and after accomplishment of the restorations.

A total of 284 laboratory orders were received, of which 33 were excluded due to various reasons (e.g., incompleteness of the registration form or no prosthodontic appliance requested, e.g., an orthodontic appliance or a diagnostic wax-up). The resulting laboratory orders represented prosthetic situations of 251 subjects (mean age 46 ± 14 years; 53% females).

### 2.2. Epidemiological Sample

To assess the representativeness of the laboratory sample, a subsample was extracted from a stratified cluster sample of a Bulgarian population in an ongoing epidemiological study (approved by the Medical University-Sofia Ethical Committee, decision no. 299). This subsample consisted of 325 subjects aged 20 years and over living in Sofia (mean age 36 ± 12 years; 34% females). Data on the state of the dentition including fixed and removable restorations were collected through dental examination after informed consent was obtained from each subject.

For each sample, proportions of crowned teeth and proportions of replaced teeth in one dental region (*a*, *p*, *m*) were calculated relative to the other two regions, that is, *a/p* + *m*, *p/a + m*, and *m/a* + *p*, where *a*, *p*, and *m* are the number of crowned or replaced teeth in anterior, premolar, and molar region, respectively. Finally, the proportions for the two samples were compared and tested for significant differences using chi-square tests.

## 3. Results

The two-week output of the 5 laboratories consisted of 243 crowns on natural teeth and implants, 16 post and cores, 18 complete dentures, and 23 removable and 82 fixed partial dentures (Table 2). The mean laboratory prices for a single crown, a three-unit fixed dental prosthesis, and an acrylic removable partial denture were *€*22, *€*75, and *€*30, respectively. Approximately half of the restorations were produced on 134 partial casts, while 203 dental restorations were produced on 115 complete casts (69 upper and 46 lower). The latter were related to the dental arch groups (Table 3). Altogether 382 teeth were replaced (167 with fixed dental prostheses and 215 with removable dentures including complete dentures) and 540 teeth received a single crown or served as a retainer. The majority of fixed dental restorations produced on partial and complete casts restored maxillary teeth. 

Two-thirds of the fixed dental prostheses (*n* = 62; 71%) in both upper and lower casts replaced posterior teeth only (Table 3). The majority of removable partial dentures delivered was acrylics (*n* = 18; 78%); 5 (22%) were metal frames. Most replacements were in the maxillary premolar region and in the mandibular molar region. There was no case of removable partial denture replacing only anterior teeth. For the slightly interrupted dental arch group, only fixed dental prostheses were produced (Table 3). All casts representing edentulous jaws received complete dentures (*n* = 18). 

Before delivery of the requested restoration, 19 sets of casts (33%) represented interrupted/reduced dentitions, 25 (43%) were slightly interrupted, and 14 (24%) were recognized as shortened dental arch conditions (Table 4). After delivery, only 19 (33%) out of 58 incomplete dentitions were restored to the level of completeness (Table 4). The majority of the incomplete dentitions resulted in slightly interrupted (*n* = 15; 26%) or shortened dentitions (*n* = 24; 41%), including 10 cases (4 shortened dentitions and 6 slightly interrupted dentitions) for which only crowns and no replacements were requested.

In the epidemiological subsample, a total of 428 missing teeth appeared to be replaced (222 with fixed dental prostheses and 206 with removable dentures including complete dentures) and 600 teeth were crowned. Fixed dental restorations were more common in the upper jaw. Approximately 2/3 of all missing anteriors and 1/2 of the missing premolars were replaced, while 2/3 of all missing first and second molars were not replaced. The laboratory sample revealed significant differences in the proportions of crowned teeth per dental region compared to the epidemiological subsample (chi-square tests; *P*  values < .0001), except for the upper molar region (*P* = .91) and the lower premolar region (*P* = .20) (Table 5). The laboratory sample showed relatively more crowns made in the anterior region than the epidemiological subsample and is therefore considered not representative for the Sofia population. In contrast, no significant differences were found between the proportions of teeth replaced (all *P*  values > .168) (Table 5).

## 4. Discussion

This study investigated prosthodontic restorations produced by 5 dental laboratories in a 14-day period and related this production to dental regions, dental arches, and dentitions as noted from the dental casts. Relating production to dentition situations before and after insertion of the produced restoration is a different approach to describe provision of prosthetic restorations that emphasise the importance of the treatment outcome rather than the production of a certain type of restoration alone. Approximately half of the laboratory production in this study, however, was produced on partial casts and a relatively small number of prosthetic restorations produced on complete casts was related to dentition situations. This diminished the sample size and therefore the findings of this study must be viewed with caution. 

Another limitation of this study is the convenient sampling of the dental laboratories. Although a random selection of laboratories would have been preferable for obtaining an unbiased estimate of prosthodontic production in Sofia, a truly representative sampling was not feasible. The main reason for this was the recording of information on consecutive work orders together with information on cost of the appliance, which provoked owners of dental laboratories to view this investigation with suspicion. It is not known to what extent the inclusion only of laboratories willing to participate influenced the results in this study. However, the output of the dental laboratories involved was considered representative for Sofia as their clientele was well known for practicing widely in the city. 

The laboratory sample represented a patient population and cannot be considered representative for the nonpatient population. The mean age and the prevalence of females in the laboratory sample were higher compared to the Sofia population sample. Higher mean age in the laboratory sample can be partly attributed to 18 subjects of 66 to 85 years of age requiring removable dentures, while the Sofia sample consisted of a working population between 20 and 65 years of age. Moreover, it has been shown that prosthodontic restorations are more common in older ages [10]. The same is true for females who seek dental treatment more often than males [11, 12]. This difference in age and gender distribution did not allow comparison of subjects, dental arches, and dentitions between the two samples. However, the Sofia population sample comprised both patient as well as nonpatient individuals. Therefore, it was considered appropriate to compare the proportions of crowned and replaced teeth per dental region in the two samples in order to verify whether number, type, and location of the produced restorations are reflected in the population. 

The distributions of crowned teeth in the two samples differed significantly. In contrast, the distributions of replaced teeth over the dental regions were similar in both samples. It can be therefore concluded that the tooth replacements as produced in the laboratories were sufficiently reflected in the Sofia population sample. More than half of the incomplete dentitions in the laboratory sample of this study were restored to the level of shortened dentitions or did not receive any replacement. In a recent similar laboratory study in a southern region of Vietnam, it was concluded that dental practitioners tend to provide complete dental arches by tooth replacements, predominantly acrylic removable partial dentures [13]. In contrast, dental practitioners in Sofia seem to direct more efforts towards preservation of present teeth (with crowns) rather than replacing absent teeth. Thus, prosthodontic output (as requested by dentists) of dental laboratories in Sofia indicates that dentists tend to provide fixed dental prostheses to restore incomplete dentitions to a functional level and not necessarily to complete dentitions. These findings are in line with contemporary treatment concepts, based on the assumption that reduced dentitions can offer sufficient oral function [14–16]. Moreover, it has been suggested that fixed rather than removable prostheses should be used even in elderly patients [17]. 

In this study, production of both fixed and removable prostheses was investigated, and results from the insertion of produced restorations were appraised on dentition level. In contrast, other studies on prosthodontic production investigated production of either fixed or removable dental prostheses, focusing on the number and the distribution of produced restorations on dental arch level (upper or lower), number and type of teeth most commonly crowned or replaced, or different technical aspects of the production process, for example, design, material or quality of the restorations [18–22]. Therefore, comparison of the present material with results of the few available studies on prosthodontic production cannot be conclusive. 

The total output of the laboratories was rather small, although the number of the laboratories and the observation period are consistent with previous surveys [13, 18, 19]. This may be a result of the limited access of the Sofia population to dental care due to financial restrictions, particularly with respect to prosthodontic treatment which is not covered at all by the national health insurance fund in Bulgaria. In 2007, the average monthly salary of employees under labor contract in Sofia city was 590 leva or approximately *€*300 [23]. This is four times the mean laboratory price of a three-unit FDP (not including the dentists' fees). It is assumed that out-of-pocket payment for a fixed dental prosthesis is a substantial effort for considerable part of the population. Despite the high costs, the production of fixed dental prostheses noticeably outnumbered the production of removable dentures. The low cost of acrylic removable partial dentures apparently did not lead to effective demand for this type of appliances.

## 5. Conclusions

Within the limitations of this study, it can be concluded that dentists in Sofia predominantly decide to provide fixed restorations. The majority of prosthetic restoration requests were not aiming at restoring incomplete dentitions to the level of completeness. Shortened and slightly interrupted dentitions appear to be acceptable treatment goals for the restoration of mutilated dentitions. Further investigation is recommended to address this topic. 

## Figures and Tables

**Table 1:** Dental arch groups and dentition groups of the complete sets of casts according to the number of teeth present and group characteristics.

Dental arch			Dentition		
Group	No. of teeth	Characteristics	Group	No. of teeth	Characteristics
Edentulous	0	(i) no teeth	Edentulous	0	(i) no teeth
			Edentulous one jaw	≥1	(i) in opposing jaw ≥1 teeth

Interrupted/reduced	≤10	(i) open space(s)*/anterior reduction	Interrupted/reduced	≥2	(i) one or both jaws ≤ 10 teeth (ii) open space(s)*/ anterior reduction

Slightly interrupted	≥11	(i) open space(s)*	Slightly interrupted	≥22	(i) in each jaw ≥11 teeth (ii) open space(s)*

Shortened	≥6	(i) anterior region intact (ii) posterior absent teeth (iii) no open space(s)*	Shortened	≥12	(i) anterior regions intact(ii) posterior absent teeth in one or both jaws (iii) no open space(s)* in occluding area

Complete	≥14	(i) with or without 3rd molars	Complete	≥28	(i) with or without 3rd molars

*An open space is defined as tooth-bounded edentulous area.

**Table 2:** Number (%) of prostheses delivered by the dental laboratories during the two-week recording.

Prostheses	Dental laboratory					Total
	1	2	3	4	5	
Fixed dental prosthesis	30 (18)	12 (26)	8 (22)	24 (24)	8 (25)	82 (21)
Removable partial denture	11 (7)	*	2 (5)	10 (10)	0	23 (6)
Crown	108 (65)	33 (72)	24 (65)	49 (47)	24 (75)	238 (63)
Implant retained crown	5 (3)	0	0	0	0	5 (1)
Post & core	8 (5)	1 (2)	3 (8)	4 (4)	0	16 (4)
Complete denture	3 (2)	*	*	15 (15)	*	18 (5)
Total	165 (100)	46 (100)	37 (100)	102 (100)	32 (100)	382 (100)

*Does not provide this service.

**Table 3:** Number of delivered crowns (C), post and cores (P&C), fixed dental prostheses (FDP), removable partial dentures (RPD), and complete dentures (CD) according to the dental arch groups before replacement.

Dental arch group (*n*)	C	P&C	FDP			RPD		CD	Total production
			Anterior only	Posterior only	Anterior & posterior	Posterior only	Anterior & posterior		
Edentulous (18)	*	*	*	*	*	*	*	18	18
Interrupted/reduced (31)	15	2	6	7	6	5	10	*	51
Slightly interrupted (26)	16	2	3	16	1	0	0	*	38
Shortened (14)	24	0	0	2	0	8	*	*	34
Complete (26)	62	0	*	*	*	*	*	*	62
Not assigned									
(i) partial casts (134)	126	12	2	37	2	*	*	*	179

Total production	243 (64)	16 (4)	11 (3)	62 (16)	9 (2)	13 (3)	10 (3)	18 (5)	382 (100)

* Not applicable.

**Table 4:** Dentition groups before and after delivery and type of appliance in case of replacement.

Dentition group before delivery	Prevalence *N* (%)	Type of replacement		Dentition group after delivery		
		FDP	RPD	Slightly interrupted	Shortened	Complete
Interrupted/reduced	19 (33)	16	10	4	10	5
Slightly interrupted	25 (43)	19	0	11	3	11
Shortened	14 (24)	5	6	0	11	3

Total	58 (100%)	40 (71%)	16 (29%)	15 (26%)	24 (41%)	19 (33%)

**Table 5:** Proportions of crowned teeth and replaced teeth in anterior, premolar, or molar regions relative to the other two regions in upper and in lower cast/jaw of the laboratory sample (Lab) and of the epidemiological sample (Epi).

Region	Upper cast/jaw		*P* value	Lower cast/jaw		*P* value
	Lab	Epi	*χ* ^2^	Lab	Epi	*χ* ^2^
Crowned teeth						
Anterior	175/186	134/244	.0003	44/135	15/207	.0001
Premolar	87/274	139/239	.0001	53/126	79/143	.205
Molar	99/262	105/273	.914	82/97	128/94	.018

Replaced teeth						
Anterior	61/173	72/169	.356	25/123	43/144	.168
Premolar	96/138	96/145	.791	46/102	49/138	.325
Molar	77/157	73/168	.540	77/71	95/92	.824
